# Why restricted mean survival time methods are especially useful for non-inferiority trials

**DOI:** 10.1177/17407745211045124

**Published:** 2021-09-23

**Authors:** Matteo Quartagno, Tim P Morris, Ian R White

**Affiliations:** MRC Clinical Trials Unit, Institute for Clinical Trials and Methodology, University College London, London, UK

Our attention was recently captured by the paper from Freidlin et al.,^1^ in which the authors investigate the conditions under which testing non-inferiority with time-to-event data defining the margin as a difference in restricted mean survival time (DRMST) leads to higher power than defining it as a hazard ratio (HR).

We agree with the authors that there is no magic in DRMST, and that it is important to clarify *when*, and *why*, one method is advantageous over the other. The authors have addressed the *when*, by providing a simulation study that indicates that DRMST is more powerful with low event rates, limited follow-up and large non-inferiority margins. This is a welcome addition to previous simulation studies that had either suggested a generalised power advantage of DRMST^2^ or shown differences but without investigating them further;^3^ however, the issue of clarifying *why* such differences arise remains.

There are few reasons why using HR may be advantageous over DRMST: first, DRMST discards data on follow-up after τ, as one of the scenarios in Freidlin et al.^1^ was designed to show. Second, it is compromised by loss to follow-up before τ. More generally, when estimated non-parametrically, DRMST is less efficient than HR, which is generally estimated through semi or fully parametric models under the proportional hazards assumption. It is therefore not surprising to see an advantage in terms of power for HR in certain settings. However, in several scenarios, and in particular with large non-inferiority margins and low event rates, conclusions are reversed so that DRMST has a power advantage, and the reasons for this phenomenon are not well understood.

We believe the concept of *non-inferiority frontiers*, which we introduced in a recent paper,^4^ helps to explain the *why*. The fundamental reason for the difference in power between DRMST and HR methods is that the null hypotheses are not the same, even if we make the non-inferiority margins match, as was done in Freidlin et al.^1^ and Weir and Trinqart.^3^ This is because the null hypotheses are actually curves in space or, as we called them, *frontiers*, rather than single points, which are simply used as assumptions for the purpose of designing a frequentist trial.

Figure 1 gives graphical intuition for this point. Figure 1(a) shows the non-inferiority frontiers corresponding to DRMST- and HR-based tests in a simulation scenario similar to the first in Freidlin et al.^1^ The dashed line represents the line of treatment equality, the hollow dot represents the expected control event rate, and the cross is the corresponding frontier point, that is, the non-inferiority margin if the expected point was correct. The turquoise (HR) frontier passes closer to the expected point than the navy (DRMST) frontier, and hence requires a larger sample size to conclude non-inferiority. A similar phenomenon happens with binary outcomes where, for low event rates, the frontier corresponding to a risk ratio margin passes closer to the expected point than one based on a risk difference margin, and hence implies larger sample sizes.

**Figure 1.**
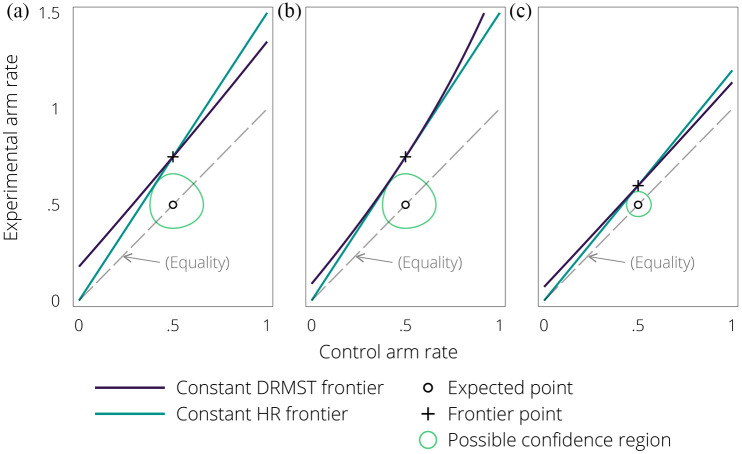
L’Abbé plot showing the non-inferiority frontiers on the DRMST and HR scale for three hypothetical trial scenarios ((a) base: moderate event fractions; large margin, (b) large event fractions and (c) small margin). The circle represents a possible confidence region for the joint distribution of control and experimental event rates for a trial for which the estimated control rate matches the expected one and the estimated active rate is as in the alternative hypothesis.

A larger event rate or a smaller margin changes the graph, as shown in Figure 1(b) and (c), respectively, so that the different frontiers are much more similar near the expected point and the other differences we listed above give HR the edge over DRMST. Estimating DRMST by fitting a Cox model could eliminate the remaining differences in favour of HR in these settings, making DRMST always at least as powerful as HR. Nevertheless, this should not be taken to mean that all non-inferiority trials should be designed using DRMST.

Since different *population-level summary measures* imply different null hypotheses, we believe the choice should be driven initially by clinical considerations and later tempered by statistical considerations. This is well recognised in certain areas: for example, for vaccine non-inferiority trials, even though, for low infection risk, defining the non-inferiority margin as a risk difference would give much greater power, the margin is usually defined as a ratio, because a relative population summary is more meaningful (and transportable) in situations where baseline risk varies. The rest of this letter details the methods we used to produce the figure.

## Details

Let λ_1_ and λ_0_ be the unknown event rates in the experimental and control arms, assumed constant. Let λ_*e*_ be the expected event rate in the sample size calculation, assumed the same in both arms. Let λ_*f*_ be the event rate in the experimental arm used to specify the non-inferiority margin: that is, if λ_0_ = λ_*e*_, then non-inferiority means λ_1_ < λ_*f*_. Thus, on the HR scale, the non-inferiority margin is λ_*f*_ / λ_*e*_. Allowing the control arm event rate to be unknown, this margin implies non-inferiority if λ_1_ / λ_0_ < λ _*f*_/ λ_*e*_. This equation relating the unknown λ_1_ and λ_0_ is the non-inferiority frontier on the constant-HR scale.

For constant event rate λ and fixed horizon τ, the restricted mean survival time (RMST) to time τ is *r*(λ) = (1 − *e* ^−λτ^) / λ. The non-inferiority frontier on this scale is *r*(λ_1_) − *r*(λ_0_) = *r*(λ_*f*_) − *r*(λ_*e*_), where *r*(λ_*f*_) − *r*(λ_*e*_) is the non-inferiority margin on the DRMST scale.

We draw the non-inferiority frontier for three settings. As a base case to illustrate the settings where DRMST shows benefit, we specify moderate event fractions and a large margin by λ_*e*_ = 0.5, λ_*f*_ = 0.75 and τ = 1. This implies the non-inferiority margin is 1.5 on the HR scale and −0.083 on the DRMST scale. We plot the non-inferiority frontiers for λ_0_ ranging from 0 to 1, with values of λ_1_ found iteratively in each case. To illustrate the settings with large event fractions, we change τ to 3. To illustrate the setting with a small margin, we change λ_*f*_ to 0.55. To graphically explain the greater distance of the DRMST frontier, we additionally show a possible confidence region for control and experimental rate from a hypothetical trial where the observed control rate matches exactly the expected one and the results are on the border of significance on the HR scale, that is, where the confidence region just touches the HR frontier; the region does not reach the DRMST frontier, hence, non-inferiority could be concluded on the DRMST scale, but not using HR.
